# A Scoping Review and a Taxonomy to Assess the Impact of Mobile Apps on Cancer Care Management

**DOI:** 10.3390/cancers15061775

**Published:** 2023-03-15

**Authors:** Eshita Dhar, Adama Ns Bah, Irene Alice Chicchi Giglioli, Silvia Quer, Luis Fernandez-Luque, Francisco J. Núñez-Benjumea, Shwetambara Malwade, Mohy Uddin, Umashankar Upadhyay, Shabbir Syed-Abdul

**Affiliations:** 1 Graduate Institute of Biomedical Informatics, College of Medical Sciences and Technology, Taipei Medical University, Taipei 106, Taiwan; 2 International Center for Health Information Technology, College of Medical Science and Technology, Taipei Medical University, Taipei 106, Taiwan; 3 Adhera Health, Inc., Palo Alto, CA 94304, USA; 4 Innovation and Data Analysis Unit, Virgen Macarena University Hospital, Andalusian Health Service, Seville 41009, Spain; fjose.nunez@juntadeandalucia.es; 5 Research Quality Management Section, King Abdullah International Medical Research Center, King Saud bin Abdulaziz University for Health Sciences, Ministry of National Guard-Health Affairs, Riyadh 11426, Saudi Arabia; 6 Faculty of Applied Sciences and Biotechnology, Shoolini University of Biotechnology and Management Sciences, Solan 173229, Himachal Pradesh, India; 7 School of Gerontology and Long-Term Care, College of Nursing, Taipei Medical University, Taipei 110, Taiwan

**Keywords:** cancer, m-health, mobile apps, health outcomes, scoping review, taxonomy, intervention, treatment

## Abstract

**Simple Summary:**

Mobile applications in clinical treatment are becoming increasingly popular among cancer patients and survivors. The COVID-19 pandemic demonstrated the importance of digital interventions in patient monitoring. We conducted a scoping review and classified Mobile Health (mHealth) trials into sub-groups based on intervention methodologies, lifestyle variables, and their effectiveness on cancer health outcomes. Our study identified the key elements of the mHealth approach for cancer care, including interactive support, personalized suggestions, active participation of users, wearable technology and rigorous theory-based solutions. We also established a taxonomy that can be employed by application developers and medical specialists in developing future mHealth cancer care solutions.

**Abstract:**

Mobile Health (mHealth) has a great potential to enhance the self-management of cancer patients and survivors. Our study aimed to perform a scoping review to evaluate the impact and trends of mobile application-based interventions on adherence and their effects on health outcomes among the cancer population. In addition, we aimed to develop a taxonomy of mobile-app-based interventions to assist app developers and healthcare researchers in creating future mHealth cancer care solutions. Relevant articles were screened from the online databases PubMed, EMBASE, and Scopus, spanning the time period from 1 January 2016 to 31 December 2022. Of the 4135 articles initially identified, 55 were finally selected for the review. In the selected studies, breast cancer was the focus of 20 studies (36%), while mixed cancers were the subject of 23 studies (42%). The studies revealed that the usage rate of mHealth was over 80% in 41 of the 55 studies, with factors such as guided supervision, personalized suggestions, theoretical intervention foundations, and wearable technology enhancing adherence and efficacy. However, cancer progression, technical challenges, and unfamiliarity with devices were common factors that led to dropouts. We also proposed a taxonomy based on diverse theoretical foundations of mHealth interventions, delivery methods, psycho-educational programs, and social platforms. We suggest that future research should investigate, improve, and verify this taxonomy classification to enhance the design and efficacy of mHealth interventions.

## 1. Introduction

Due to technological advancements and superior treatment interventions, individuals diagnosed with cancer are now living longer [1]. However, the COVID-19 pandemic has had a significant impact on cancer care, causing delays in diagnosis and treatment for many patients [2]. As the global population grows and ages, the burden of cancer continues to increase [3]. Recent data shows that approximately 10 million lives were lost due to cancer in 2020, excluding non-melanoma skin cancer. Despite the progress in cancer research and treatment, cancer remains a major health challenge and one of the leading causes of mortality worldwide [3]. Hence, there is an urgent need to strengthen long-term supportive care services as the cancer population grows [4]. Currently, cancer patients and survivors face a daunting amount of responsibilities and information related to managing their illnesses and recovery. To address this challenge, healthcare providers must equip patients with the skills and knowledge required to effectively self-manage their conditions. This includes problem-solving, decision-making, resource utilization, coordination with healthcare providers, and taking appropriate actions to promote their own health and wellbeing [5].

While traditional face-to-face interventions have been beneficial in cancer care [6,7], they are not always feasible due to a lack of services, financial coverage, distance, or incapacity [8]. Fortunately, digital interventions can help overcome this challenge, as the rapid rise of mobile technology has made psychological interventions accessible to a much wider population of survivors [9,10]. The use of short message service (SMS) and applications (apps) through mobile devices such as smartphones and tablets in the health domain is referred as mHealth [11]. Further, mHealth technology has already proved its importance in the management of cancer patients, particularly in the areas of supportive care and follow up [12,13].

Mobile apps offer several benefits, including the ability to gather self-reported measures, providing user-friendly experiences, tools for managing personal health, immediate access to vital data, and reducing potential research biases [14,15]. Moreover, real-time mHealth apps offer more personalized care by providing relevant healthcare information at a low cost, and they encourage patients to meet the goals established by healthcare professionals [16,17]. Combining an app with human supervision can significantly increase patient engagement, while also improving cancer health outcomes [18,19].

Due to its complex and multi-faceted nature [20], defining a proposed mHealth intervention and evaluating its effects requires a clear and precise taxonomy [21]. A taxonomy is a useful tool for creating classifications based on relationships and has the aim of enhancing conceptual understanding and predictions [21]. The development of a taxonomy is an ongoing process that necessitates continual feedback and modification from users [21]. Theoretical foundations have important value for categorizing the assessment of mHealth intervention outcomes [22,23,24]. The health and well-being of cancer survivors are significantly affected by various factors, such as different delivery mechanisms, social media influence, and psycho-educational programs [24]. Several types of interactive health communications, including social networking, have already been used as classification methods [21]. After integrating a scoping review with taxonomy, it could support and influence future research in this field by identifying and addressing gaps, inadequacies, and trends in the existing evidence [25].

The primary objectives of this review were: (1) to explore published studies that used mobile-based interventions among cancer patients, (2) to investigate research trends and provide recommendations for the adherence and usage of mobile-app-based interventions for cancer care management, and (3) to examine the effectiveness of mobile-app-based interventions on cancer health outcomes. Hence, this analysis classified mHealth studies into various subcategories depending on the type of intervention strategies, lifestyle factors, and their impact on health outcomes for diverse cancer types and its usage rate or adherence for cancer care management. Additionally, the secondary objective of this review was to develop a taxonomy of mobile-app-based interventions for app developers and healthcare researchers that could assist in the development of future mHealth cancer care solutions.

## 2. Materials and Methods

In this study, a scoping review was conducted to identify articles that implemented the following search strategy, study selection and data extraction method, and fulfilled the selection criteria outlined below. The studies were classified based on the methods and clinical outcomes.

### 2.1. Search Strategy

The search strategy followed the Preferred Reporting Items for Systematic Reviews and Meta-Analyses for Scoping Review (PRISMA-ScR) guidelines [26]. We searched for related studies in different online databases, including PubMed, Scopus, and EMBASE, published between 1 January 2016 and 31 December 2022. The search for the studies was conducted from 1 October 2022 to 31 December 2022. The authors performed a rigorous search based on medical subject headings (MeSH) terms and relevant publication text keywords that had been identified beforehand. These search terms included ‘Mobile application’ or ‘mHealth’ or ‘Mobile apps’; ‘Cancer’ or ‘Cancer survivors’ or ‘Neoplasms’; ‘Intervention’ or ‘Treatment’.

**Figure 1:**
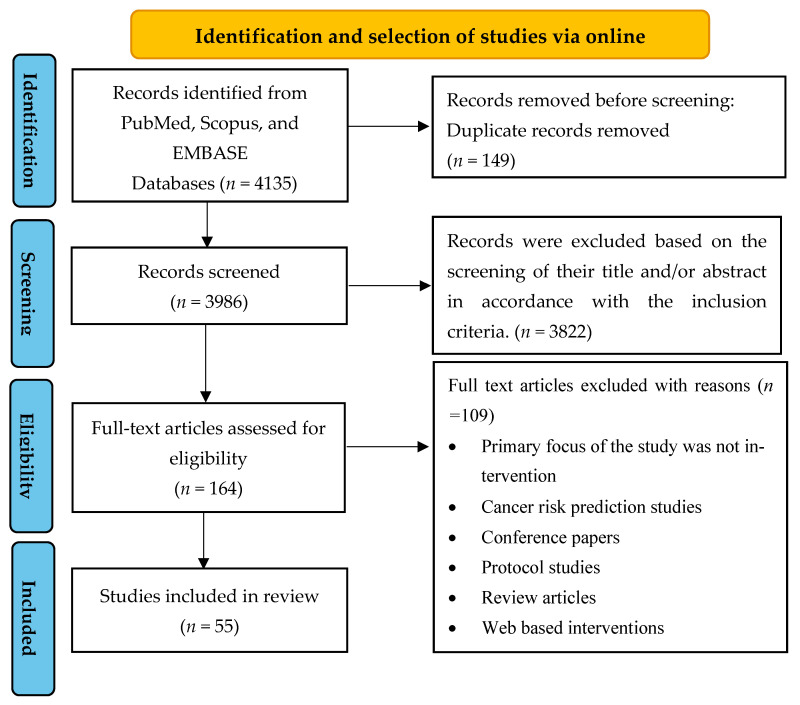
Preferred Reporting Items for Systematic Reviews and Meta-Analyses for Scoping Review (PRISMA-ScR) flow chart of study identification and selection of studies.

The detailed search strategy and results are shown in Figure 1.

### 2.2. Eligibility Criteria

The following selection criteria were applied for the inclusion of articles: (1) a focused on cancer survivors and cancer patients undergoing treatment, (2) assessed lifestyle and psychological interventions using mobile apps, (3) utilized apps for assisting patients or survivors in self-managing their health on a routine basis, (4) contained one of the following design types: randomized controlled trial (RCT), pilot study, prospective clinical trial, quasi-experimental study, feasibility study, observational or pre-test post-test study, (5) included only original research, and (6) were written in the English language only.

Studies were excluded based on the following criteria: (1) aimed at preventing or detecting cancer, (2) used telecommunication technologies such as websites, telephones, or wearables alone, (3) did not assess lifestyle or psychosocial factors or engagement with mobile apps, (4) did not focus on intervention, (5) focused on the design, development, or usefulness of mobile health apps, (6) were review articles, trial protocols, trial registrations, conference papers, book chapters, notes, brief reports, letters, editorials, case studies, and (7) were written in non-English languages.

### 2.3. Study Selection and Data Extraction

**Figure 2:**
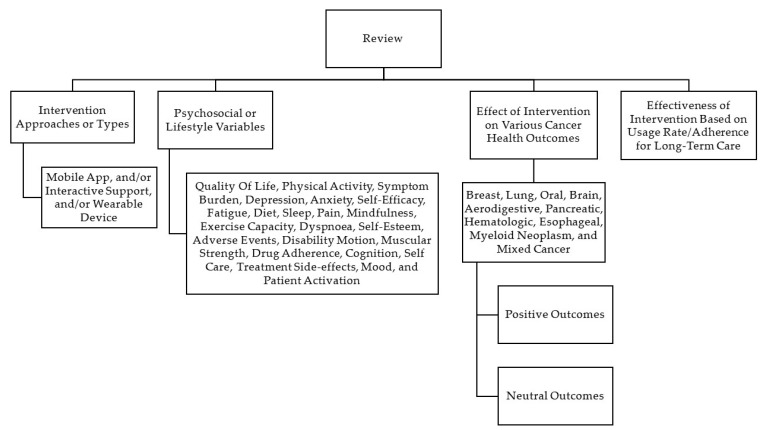
Categorization of studies included in the scoping review based on methods and outcomes.

Two authors of this study (ED and NS) conducted an independent review of the titles and abstracts of the entire search yield to identify eligible articles. If an article was considered potentially significant by either reviewer, the complete text of the publication was retrieved. In the event of a disagreement, a third reviewer (AC) decided the final article based on the inclusion and exclusion criteria. All duplicate articles were removed. An initial screening was conducted based on the titles and abstracts to identify articles that fulfilled the inclusion criteria. For those that could not be rejected with certainty, full-text articles were obtained. The authors then reviewed the full-text versions of each article to identify those that met the inclusion criteria. Subsequently, the following information was systematically extracted from each included study: study characteristics (country of origin, year of publication, and sample size), patient characteristics (mean age, gender, and type of cancer), intervention characteristics (duration, mobile app with or without interactive support and/or wearables), and intervention focus (physical and psychosocial/lifestyle variables). The selected studies were categorized based on their methods and outcomes, as shown in Figure 2.

### 2.4. Taxonomy

Our taxonomy was classified on the basis of dimensions that could potentially represent the key characteristics of users’ interaction and engagement with the interventions delivered through mobile devices.

**Figure 3:**
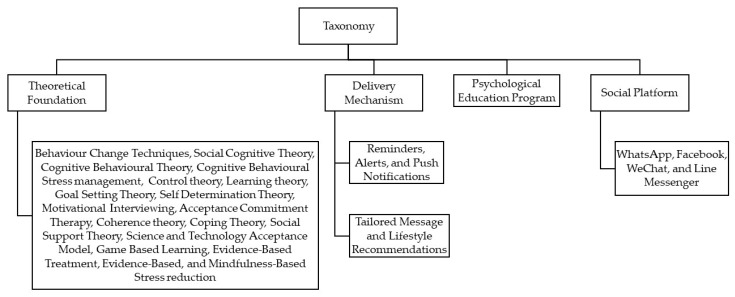
Flow of classification of the studies via taxonomy.

The four dimensions of this taxonomy are shown in Figure 3 and include the classification of mHealth interventions according to

1. Theoretical foundation or behavioural techniques [22,23,24].
2. Delivery mechanism (through reminders/alerts or tailored messages/lifestyle recommendations) [24].
3. Psycho-educational program [24].
4. Various social platforms [21,24].

## 3. Results

### 3.1. Scoping Review

#### 3.1.1. Study Selection

The electronic databases’ search yielded a total of 4135 articles from PubMed (*n* = 564), EMBASE (*n* = 587), and Scopus (*n* = 2984). After removing duplicates, 3986 articles were assessed based on their titles and abstracts. Among these, 3822 were excluded because they did not meet the inclusion criteria exclusively. The remaining 164 articles underwent full-text screening, and 109 were subsequently excluded for different reasons, such as being non-intervention studies, cancer risk prediction studies, conference papers, protocol studies, review articles, and web-based interventions (see Figure 1). Ultimately, a total of 55 studies were included in this analysis.

#### 3.1.2. General Characteristics of the Studies

Out of the fifty-five studies, nineteen were conducted in North America (eighteen in United States and one in Canada), eighteen in Asia (nine in South Korea, two each in Iran, Turkey, China, and Taiwan, and one in Japan), fourteen in Europe (three in Sweden, two each in Switzerland and Spain, and one each in United Kingdom, Ireland, Denmark, Norway, Netherlands, Slovenia, and Germany), two in Australia (Australia and New Zealand), and one in South America (Brazil). One study was conducted at multiple centres, with study participants recruited across five European nations, namely Austria, Greece, Ireland, Norway, and the UK.

In terms of study the designs of the selected studies, there were thirty-five randomized controlled trials, six feasibility studies, five pilot studies, three quasi-experimental studies, two pre-post studies, and one each of baseline/post study, prospective clinical trial and randomized open-label trial (see Table 1).

#### 3.1.3. Characteristics of Research Participants

Most of these articles targeted breast cancer only (20 studies) or breast cancer and other types of cancer (denoted by the term mixed cancer, 23 studies). Two studies focused on each of the following: prostate cancer, pancreatic cancer and lung or/and non-small cell lung cancer. The remaining six studies focused on oral cancer, brain tumour, aerodigestive cancer, myeloid neoplasm, hematologic cancer and oesophageal cancer. The number of participants across the 55 studies varied between 10 and 829. The mean age of participants ranged from 14.2 years to 72 years. The duration of mobile-app-based interventions varied between 4 and 32 weeks. The current review included studies with both younger and older cohorts. Paediatric and adolescent cancer studies [27,28,29,30] accounted for four of the fifty-five cancer trials. The remaining 51 studies focused on adults and older patients.

#### 3.1.4. Measurement Tools

Subjective self-reported questionnaires and electronic patient-reported outcome measures that had previously been validated or applied in cancer research were used in all of the included studies.

### 3.2. Categorisation of Studies

The studies included in our analysis were categorized based on their interventional approaches and clinical outcomes (see Figure 2). The categories were classified as below.

#### 3.2.1. Interventional Approaches/Types

All studies in our analysis used various intervention methods to improve cancer health outcomes. Of these, twenty-six studies solely relied on mobile-based interventions, seventeen studies used mobile-based interventions with interactive support, six studies utilized mobile-based interventions with wearable devices, and six studies combined mobile-based interventions with wearable devices and interactive support (see Table 1).

The mobile-based interventions in the studies monitored cancer health outcomes while providing motivational texts, educational support, coping skills training, and game-based learning. When integrated with wearable devices, the mHealth apps collected real-time data and offered feedback. The interventions with interactive support were provided by healthcare professionals, qualified counsellors, or researchers, and involved personalized assistance, treatment, coaching, guidance, counselling, and motivation delivered via phone calls, mobile apps, or face-to-face interactions.

#### 3.2.2. Psychosocial/Lifestyle Variables Assessed

There were forty-three studies that assessed quality of life, fifteen studies that focused on physical activity, ten studies that targeted anxiety, nine studies that addressed symptom burden and management, seven studies each for fatigue, loneliness/depression, nutrition/diet, and self-efficacy, six studies for sleep quality, and five studies each for pain and mindfulness. Additionally, three studies investigated exercise capacity, two studies examined smoking cessation/abstinence, and one study each focused on dyspnoea, adverse events, disability motion, muscular strength, drug adherence, cognition, self-care activities, patient activation (self-management of illness), treatment side effects, self-esteem, and utilization of supportive care services. These variables have been listed under the ‘focus of the study’ in Table 1.

#### 3.2.3. Effects of Interventions on Various Cancer Health Outcomes

The term “positive outcomes” referred to any improvement in participants’ health as a result of using mobile apps. Positive outcomes were found in 19 of 20 breast cancer studies, 19 of 23 mixed cancer studies, two studies each for prostate and lung cancer, and one study each for oral, brain, pancreatic, and aerodigestive cancer. Neutral outcome implied no significant improvement in the health outcomes of the participants and was seen in nine studies [27,29,30,31,32,33,34,35,36]. Improvements in cancer health outcomes are summarized in Table 1.

**Table 1:** Characteristics of included studies and mobile-based interventions.

Author, Year, Country	Cancer Type	Sample Size	Study Design	Gender (%) Mean Age (Years)	Mobile App and/or Interactive Support and/or Wearables Device	Study Focus	Study Duration	Improvements in Health Outcomes
Egbring et al., 2016 [18] Switzerland	Breast Cancer	139	RCT	100% females 53 (yrs)	Mobile app + interactive support	Daily functional activity and adverse events	6 weeks	Daily functional activity and patient awareness of severity of symptoms
Lozano et al., 2019 [37] Spain	Breast Cancer	80	Prospective test-retest quasi-experimental study	100% females 51.80 (yrs)	Mobile app only	QoL, physical activity, body composition and physical activity motivation	8 weeks	QoL, physical activity and body weight
Allicock et al., 2021 [38] USA Ballcock	Breast Cancer	22	Feasibility study	100% females 52.23 (yrs)	Mobile app + wearable device	Physical activity and diet	8 weeks	Physical activity and diet habits
Yanez et al., 2020 [39] USA	Breast Cancer	78	RCT	100% females 52.54 (yrs)	Mobile app + interactive support	QoL, symptom burden (breast cancer related)	6 weeks	Breast cancer well-being (disease specific QoL) and symptom burden
Cinar et al., 2021 [40] Turkey	Breast Cancer	64	RCT	100% females 45.7 (yrs)	Mobile app + interactive support	QoL, distress	12 weeks	QoL and distress
Handa et al., 2020 [29] Japan	Breast Cancer	95	RCT	100% females 49.9 (yrs)	Mobile app only	QoL (Anxiety and depression), health literacy	12 weeks	No improvement
Uhm et al., 2017 [16] South Korea	Breast Cancer	339	Quasi-experimental study	100% females 50.3 (yrs)	Mobile app + wearable device	QoL, physical measurements and self-reported physical activity	12 weeks	QoL and physical activity
XU et al., 2021 [41] China	Breast Cancer	126	RCT	100% females 47.93 (yrs)	Mobile app + interactive support	QoL (anxiety and depression), discomfort symptoms and self-efficacy	16 weeks	QoL and self-efficacy
Ghanbari et al., 2021 [42] Iran	Breast Cancer	82	RCT	100% females 46.45 (yrs)	Mobile app + interactive support	Anxiety and self-esteem	4 weeks	Anxiety and self-esteem
Sheean et al., 2021 [43] USA	Breast Cancer	35	RCT	100% females 55.11 (yrs)	Mobile app + interactive support	QoL, symptom burden, lifestyle behaviours (nutrition and physical activity)	12 weeks	QoL and physical activity
Kuhar et al., 2020 [44] Slovenia	Breast Cancer	91	Non-randomized controlled prospective cohort Study	100% females 51.7 (yrs)	Mobile app only	QoL	Throughout chemotherapy	QoL
Lozano-Lozano et al., 2020 [45] Spain	Breast Cancer	78	RCT	100% females 52.5 (yrs)	Mobile app only	QoL, disability, motion, muscular strength	8 weeks	QoL, disability, motion and muscular strength
Kim et al., 2018 [46] South Korea	Breast Cancer	72	RCT	100% females 51 (yrs)	Mobile app only	QoL, drug adherence, side effects depression and anxiety	3 weeks	QoL, compliance to medication and side effects
Rosen et al., 2018 [47] USA	Breast Cancer	84	RCT	100% females 53 (yrs)	Mobile app only	QoL and mindfulness	8 weeks	QoL and mindfulness
Lengacher et al., 2018 [48] USA	Breast Cancer	13	Pilot Study	100% females 57 (yrs)	Mobile app only	QoL, fatigue, depression, pain, sleep quality, stress, FOR, anxiety, cognition and mindfulness	6 weeks	QoL, fatigue, depression, pain, sleep quality, anxiety, stress, FOR, anxiety, cognition and mindfulness
Ferrante et al., 2020 [49] USA	Breast Cancer	35	RCT	100% females 61.54 (yrs)	Mobile app + wearable device	QoL, weight management, diet and physical activity, cardiopulmonary fitness, social cognitive theory variables and anthropometric outcomes	24 weeks	QoL, waist circumference, healthy eating and calorie reduction techniques
Hou et al., 2020 [50] Taiwan	Breast Cancer	112	RCT	100% females 42 (yrs)	Mobile app only	QoL	12 weeks	QoL
Fjell et al., 2020 [51] Sweden	Breast Cancer	149	RCT	100% females 49 (yrs)	Mobile app + interactive support	QoL and symptom burden	18 weeks	QoL and symptom burden
Öztürk et al., 2021 [52] Turkey	Breast Cancer	57	RCT	100% females 51.44 (yrs)	Mobile app only	QoL and symptom burden	8 weeks	QoL and symptom burden
Bandani-Susan et al., 2021 [53] Iran	Breast Cancer	38	RCT	100% females 46.34 (yrs)	Mobile app only	Cancer-related fatigue	7 weeks	Fatigue
Mendoza et al., 2017 [27] USA	Mixed Cancer	59	RCT	59.3% females 16.6 (yrs)	Mobile app + wearable device + interactive support	QoL and physical activity	10 weeks	No improvement
Blair et al.,2021 [54] USA	Mixed Cancer	54	RCT	55% females 69.6 (yrs)	Mobile app + wearable device + Interactive support	QoL and physical activity	13 weeks	Physical activity
Kubo et al., 2019 [55] USA	Mixed Cancer	97	RCT	69% females 59 (yrs)	Mobile app only	QoL, fatigue, distress, sleep, mindfulness, pain, anxiety and depression, and posttraumatic growth	8 weeks	QoL
Puszkiewicz et al., 2016 [56] UK	Mixed Cancer	11	Pre–post study	82% females 45 (yrs)	Mobile app only	QoL, physical activity, well-being, fatigue, sleep, anxiety and depression	6 weeks	Physical activity and sleep quality
Yang et al., 2019 [57] China	Mixed Cancer	58	RCT	34% females 52.53 (yrs)	Mobile app only	QoL and pain	4 weeks	QoL and pain
Mikolasek et al., 2021 [58] Switzerland	Mixed Cancer	100	Feasibility study	74% females 53.2 (yrs)	Mobile app only	QoL, anxiety, fatigue, distress, sleep and mindfulness	20 weeks	QoL, anxiety, fatigue, distress, sleep disruptions and mindfulness
Walsh et al., 2021 [59] Ireland	Mixed Cancer	123	RCT	86% females 57.42 (yrs)	Mobile app + Wearable device	QoL, fatigue, self-efficacy, loneliness, exercise self-efficacy, social support for physical activity, functional exercise capacity, diet and physical activity	12 weeks	BMI, waist circumference and physical activity
Poort et al., 2021 [30] USA	Mixed Cancer	25	Pilot study	56% females 28 (yrs)	Mobile app only	QoL, self-efficacy for coping with cancer, self-efficacy for managing emotions and perceived emotional support	12 weeks	No improvement
Robertson et al., 2020 [60] USA	Mixed Cancer	78	RCT	91% females 55.1 (yrs)	Mobile app + wearable device	Physical activity	16 weeks	Physical activity
Pappot et al., 2019 [12] Denmark	Mixed Cancer	20	Pre–post study	70% females 25 (yrs)	Mobile app only	QoL	6 weeks	QoL
Jibb et al., 2017 [28] Canada	Mixed Cancer	38	One-group baseline/poststudy	43% females 14.2 (yrs)	Mobile app only	QoL, pain and self-efficacy	4 weeks	QoL and pain
Livingston et al., 2020 [31] Australia	Mixed Cancer	82	RCT	71% females 59.5 (yrs)	Mobile app only	Unmet psychological needs	16 weeks	No improvement
Børøsund et al., 2020 [61] Norway	Mixed Cancer	25	Feasibility study	84% females 48 (yrs)	Mobile app + interactive support	QoL, anxiety and depression, fatigue, stress	8 weeks	QoL, anxiety, fatigue and stress
Ham et al., 2019 [62] South Korea	Mixed Cancer	63	RCT	86% females 44.1 (yrs)	Mobile app only	QoL, depression and anxiety	10 weeks	Depression and anxiety
Benze et al., 2019 [63] Germany	Mixed Cancer	40	Feasibility study	70% females 57 (yrs)	Mobile app only	QoL, pain, distress and symptoms	24 weeks	QoL and symptom burden
Greer et al., 2019 [64] USA	Mixed Cancer (high anxiety)	145	RCT	74% females 56.45 (yrs)	Mobile app only	QoL, anxiety and depression	12 weeks	QoL, anxiety and depression
Maguire et al., 2021 [65] Austria, Greece, Ireland, Norway and UK	Mixed Cancer	829	RCT	82% females 52.4 (yrs)	Mobile app + interactive support	QoL, symptom burden, supportive care needs, work limitations, anxiety and self-efficacy	18 weeks	QoL, symptom burden, anxiety and self-efficacy
Ormel et al., 2018 [66] Netherland	Mixed Cancer	32	RCT	13% females 33.6 (yrs)	Mobile app only	Physical activity	12 weeks	Physical activity
Krebs et al., 2019 [67] USA	Mixed Cancer	38	RCT	71% females 57.11 (yrs)	Mobile app + interactive support	Smoking cessation	4 weeks	Higher confidence to quit
Casillas et al., 2019 [68] USA	Mixed Cancer	71	RCT	53% females 21 (yrs)	Mobile app + interactive support	Survivorship care knowledge and self-efficacy	8 weeks	Survivorship care attitude and self-efficacy
Rico et al., 2020 [69] Brazil	Mixed Cancer	87	RCT	56% females 45.2 (yrs)	Mobile app + interactive support	QoL side effects	12 weeks	QoL (side effects)
Chung et al., 2022 [70] South Korea	Mixed Cancer	41	RCT	80% females 41.78 (yrs)	Mobile app only	QoL and sleep quality	10 weeks	Sleep quality
Merz et al., 2022 [33] USA	Mixed Cancer	45	RCT	60% females	Mobile app only	QoL, utilization of supportive care services and patient activation (self-management of illnesses)	12 weeks	No Improvement
Sundberg et al., 2017 [71] Sweden	Prostate cancer	130	Feasibility study	100% males 69 (yrs)	Mobile app + interactive support	QoL and symptom burden	5–8 weeks	QoL and symptom burden
Lee at al., 2019 [72] South Korea	Prostate cancer	100	Randomized open-label trial	100% males 69.44 (yrs)	Mobile app + wearable device+ interactive support	Physical functions	12 ± 1 weeks	Physical functions
Ji et al., 2019 [73] South Korea	Non-small cell lung cancer	64	Prospective clinical trial	70% males 59.23 (yrs)	Mobile app + wearable Device	QoL, exercise capacity and dyspnea	12 weeks	QoL, exercise capacity and dyspnea
Park et al., 2019 [74] South Korea	Lung cancer	90	Pilot study	46% males 55.1 (yrs)	Mobile app + wearable + interactive support	QoL, exercise capacity and symptom management	12 weeks	Exercise capacity and symptom management.
Wang et al., 2020 [75] Taiwan	Oral cancer	100	Quasi experimental	92% males 57.01 (yrs)	Mobile app + interactive support	QoL (symptoms), cancer needs	12 weeks	QoL and cancer care needs
De Tommasi et al., 2020 [76] New Zealand	Brain tumour	10	Feasibility study	60% females 53.8 (yrs)	Mobile app only	QoL (illness-related), psychological distress and mindfulness capacity	8 weeks	QoL and mindfulness
Rettig et al., 2018 [77] USA	Aerodigestive cancer	29	RCT	62% males 55 (yrs)	Mobile app + interactive support	Smoking abstinence	8 weeks	Smoking abstinence
Gustavell et al., 2019 [32] Sweden	Pancreatic and Periampullary cancer	26	RCT	61% males 66.5 (yrs)	Mobile app only	QoL, self-care activity	32 weeks	No Improvement
Keum et al., 2021 [78] South Korea	Pancreatic cancer	33	RCT	63% males 61.5 (yrs)	Mobile app + interactive support	QoL and nutrition	12 weeks	QoL and nutrition
Chow et al., 2021 [19] USA	Hematologic cancer	41	RCT	48.8% females 45.1 (yrs)	Mobile app + wearable + interactive support	QoL, physical activity, self-efficacy and diet	16 weeks	No improvement
Loh et al., 2022 [35] USA	Myeloid neoplasm	22	Pilot study	68% males 72 (yrs)	Mobile app + wearable + interactive support	QoL, physical activity, fatigue and mood	8–12 weeks (two cycles of chemotherapy)	No improvement
Yang et al., 2021 [36] South Korea	Oesophageal cancer	30	Pilot study	100% males 59 (yrs)	Mobile app + interactive support	Physical activity and nutrition	8 weeks	No improvement

QoL, quality of life; RCT, randomized controlled trial; yrs, years; FOR, fear of recurrence.

#### 3.2.4. Effectiveness of Mobile-App Based Interventions Based on the Usage Rate/Adherence for Cancer Care Management

To comprehend the general trends for app adherence or usage rate in cancer care management, we tracked the participants’ usage of the applications and positive/neutral outcomes across all cancer-type studies (see Table 2).

There is no established standard measure or threshold for defining good or bad adherence rates in app-based interventions for cancer care [79]. Adherence or usage rate was evaluated using various criteria outlined in the selected articles. For the majority of the included studies, we assessed adherence/usage rate based on data collection completeness, which refers to the percentage of enrolled patients who completed the research. The remaining studies had their own specific criteria for evaluating adherence. The study’s usage rate was defined as the percentage of enrolled users who used the app as intended, based on data logging of the application or until the end of the intervention [38,73]. Nearly 70% of the total studies included in this scoping review reported an 80% usage rate. Therefore, we determined 80% as a suitable threshold for comparing the usage rates of all the included studies. Of the included studies, 41 indicated an adherence rate of 80% or greater, while 14 reported less than 80% adherence (see Table 3). One study had a notably high dropout rate of 60%, despite having a short intervention period of only four weeks. This was attributed to the participants’ advanced age and lack of experience with the game-based app [67].

Based on our analysis, the definition of dropouts varied across studies. Some studies considered users who logged in only once or used the app once or twice as dropouts [79,80], while another study set a minimum weekly usage of 90 min [39]. Therefore, for the present investigation, the dropout rate was defined as the percentage of users who stopped using the mHealth app [80]. The review presented usage rate and study duration results for each cancer type (see Table 2).

### 3.3. Taxonomy

Out of the fifty-five studies, forty-nine were classified based on the proposed taxonomy’s four different dimensions. However, six studies could not be categorized according to the taxonomy as they did not fit into any of its dimensions [12,18,19,33,51,77]. The classification of articles based on the four dimensions of the taxonomy outlined in Figure 3 is presented in Table 4.

#### 3.3.1. Theoretical Foundations or Behavioural Techniques

A total of 22 studies utilized a theoretical foundation to encourage behavioural engagement in their mobile-based interventions. Among these studies, the most commonly used theory was social cognitive theory (SCT), which was applied in seven studies, followed by behaviour change techniques (BCTs) in five studies. Additionally, control theory (CT), learning theories (LTs), and goal setting theory (GST) were implemented in three studies each.

One study by Lozano et al. [37] incorporated a variety of BCTs, such as reinforcement, facilitation, self-monitoring, goal setting, performance feedback, and goal review. By incorporating cognitive behavioural therapy (CBT) strategies, some studies used stress management techniques, relaxation training, behavioural activation, cognitive restructuring, problem solving approaches, activity planning, and pacing, along with techniques for generating new thoughts, staying present, summarizing, and reviewing [61,62,64,70].

#### 3.3.2. Delivery Mechanism (Reminders/Alerts or Tailored Messages/Lifestyle Recommendations)

The studies included in the current taxonomy have been classified based on their intervention methods, which include reminders/alerts/notifications and personalized lifestyle recommendations/tailored messages. Out of the total 20 mHealth studies, alerts, reminders, or push notifications were used as a means of intervention. These interventions led to improved quality of life (QoL), pain-related outcomes, activity levels, anxiety, fatigue, symptom burden, exercise ability, sleep, stress, disability motion, muscular strength, mindfulness, and cancer care needs. However, only two studies failed to show any significant improvements [31,34].

On the other hand, thirteen studies provided personalized recommendations or tailored messages as their means of intervention, which motivated cancer patients to change their lifestyle habits or achieve their goals. These interventions resulted in improvements in various aspects of health, such as QoL, physical activity, symptom burden, exercise capacity, dyspnoea, smoking, self-efficacy, diet, sleep, weight, and even survivorship care attitudes. However, four studies did not show any significant improvements [27,29,35,36].

#### 3.3.3. Psychoeducational Program

Seven studies included psychoeducation as a component of their interventions for cancer patients. These apps provided information on cancer, exercises, balanced diet, and therapeutic interventions for managing fatigue, pain, sleep, anxiety, depression, self-esteem, and stress.

#### 3.3.4. Various Social Platforms

Five studies utilized social media platforms to provide cancer care services. Platforms such as WeChat app, Facebook, WhatsApp, and LINE app enabled patients to join groups and interact with each other while also receiving support and guidance from a healthcare provider. These studies demonstrated the potential of social media in facilitating peer support and improving patients’ psychological and social well-being.

## 4. Discussion

### 4.1. Summary and Findings

The objective of this review was to examine the characteristics of intervention studies and evaluate the impact of mobile health technologies on cancer health outcomes, adherence, and usage rates among cancer patients. This review analysed 55 studies that utilized mobile technology to enhance psychosocial or lifestyle habits in cancer patients and survivors. The studies included various cancer populations, age groups, mHealth interventions, and cancer outcome measures. The secondary objective was to develop a taxonomy based on mobile applications and interventions. All the studies were classified into four distinct categories: theoretical foundation, delivery mechanism, psycho-educational programs, and various social platforms. Despite the diverse range of studies included in the review, the results demonstrated that mobile health interventions were effective and well-received.

Several interventional strategies were employed to enhance cancer health outcomes. Although cancer survivorship is characterized by persistent physical and psychological challenges that make lifestyle modifications and management more challenging, the majority of these interventions had a favourable impact on cancer health outcomes [59].

In 32 of 43 trials, mHealth treatments enhanced at least one component of cancer patients’ quality of life. This is consistent with another meta-analysis that also reported improvements in quality of life among cancer patients [82]. Of the seven psycho-educational interventions based on mHealth, three resulted in an improvement in quality of life. In contrast, a meta-analysis found that internet-based psycho-educational treatments reduced depression and fatigue but had no effect on distress or quality of life [75].

Out of a total of 15 studies focused on physical activity, 11 reported a positive impact on patients’ physical activity levels. Similarly, another systematic review found that mobile applications had a positive impact on physical activity among cancer patients, utilizing various theoretical frameworks. The only difference was that this particular review focused specifically on the effectiveness of gamification interventions for improving cancer health outcomes [83].

Our review included several studies, which indicated that symptom management was improved in 7 out of 9 studies, and anxiety levels were enhanced in 7 out of 10 studies. These results align with another review that emphasized the beneficial effects of digital interventions on anxiety and symptom management. However, the latter review encompassed a wider range of interventions, such as web-based platforms, mobile apps, tele monitoring, and telemedicine in cancer care, whereas our review focused only on mobile-based interventions for cancer care [84].

Several studies reported that testing the effectiveness of their intervention was challenging due to a small sample size [28,30,32]. One study identified a communication barrier between cancer patients and healthcare professionals, which negatively impacted the patients’ health outcomes. The report suggested that if healthcare providers had offered additional support, the patients would have used the app more frequently [30].

Previous studies have shown that high drop-out rates pose a significant challenge to the success of digital health initiatives in terms of improving adherence and providing support and follow-up [85]. Our review found that the duration of the research did not have a significant impact on the adherence rate of most mobile applications. While mobile devices may appear to be a practical solution in healthcare, users may initially use them but then fail to continue using them regularly over time [31]. For instance, two trials included in our review showed that elderly users lacked familiarity with the mobile devices, resulting in drop-out rates of 59% and 60% [32,68]. One study from our review attributed its 55.2% completion rate to one-way communication, which they believed reduced patient engagement [69]. This is consistent with a review that found that two-way text messaging improves medication adherence [86]. Our review also identified other factors that could affect the effectiveness of mobile-based interventions, such as small sample size, paper-based questionnaires, and the absence of a control group [30,31,59,61]. Lower usage rates were also associated with how data was obtained, with objective measures (such as number of log-ins) being more reliable than self-reported questionnaires [37]. The reasons for dropouts in our review included aggravated sickness, old age, technical challenges with devices/apps, fatigue, mortality, personal reasons, lack of intervention support, and unfamiliarity with the mobile device. In our review, three studies examined approaches to addressing the problem of attrition [31,62,67]. These studies suggested that a blended healthcare delivery model, which combines mobile interventions with face-to-face consultation or telephone support, could be effective. Additionally, time flexibility was found to be essential for cancer survivors to fully benefit from mHealth solutions. The studies also recommended providing a variety of interventions, such as survivorship features and different exercises, to address this issue.

After conducting the review, several significant themes emerged from analysing various mHealth solutions. As a result, we have put forward a taxonomy that categorizes all treatments based on their theoretical foundations, delivery methods, psycho-educational tools, and social platforms. Whilst mHealth treatments have huge amount of potential, it is important to develop them using a theory and evidence-based methodology [24,59]. BCTs help in designing and presenting difficult treatments in a systematic way [59]. From the analysis, we discovered that BCTs can potentially lead to favourable lifestyle modifications [38,46,56,60,67]. Some of these techniques used in the apps included goal setting (behaviour), action planning, performance feedback, self-monitoring, instructions on how to perform behaviour, graded tasks, prompts and cues, or social reward [37,56,59]. Besides this, patients in the included studies took an active role in managing their own health, which may have resulted in a rise in the usage of supportive care services. Self-management strategies such as psycho-education therapies, exercise programs, and (online) self-care interventions have shown to promote patient activation by giving them knowledge and training problem solving and coping skills [80]. In three of the included studies, gamified apps were also implemented to improve cancer health outcomes [47,60,68].

We discovered that nearly 84% of the articles included in our review showed a positive response, which is consistent with prior reviews that have demonstrated improvements in cancer health outcomes [87,88,89]. It is possible that this is because the trials in our investigation had access to self-monitoring tools as well as automated sensors, online social support, and, most importantly, real-time feedback systems.

Previously, a taxonomy was developed to distinguish various technology modalities in clinical applications [90]. Furthermore, the credible classification of treatments in terms of BCTs exists, as indicated in prior studies [22,90,91,92]. Based on existing related work [23,93], we propose a framework for mobile-based interventions in cancer care. In our taxonomy, classification is conducted based on four dimensions: theoretical foundations, delivery mechanisms, psychoeducational materials, and social support via social media.

The widespread use of social media platforms such as Twitter, Facebook, and YouTube, as well as online support, represents a significant opportunity for mHealth apps in cancer survivorship [24,94]. In our study, three out of four studies successfully implemented mHealth solutions with social networks, demonstrating improvements in quality of life and self-esteem, and decreased cancer care needs. Our findings suggest that online mobile-based solutions can effectively enhance psychosocial and quality of life outcomes, while also reducing anxiety levels in cancer patients [95]. These results are consistent with Attai et al. [96], who found that participating in a Twitter support group increased cancer knowledge and decreased anxiety levels among patients.

### 4.2. Study Strengths

Our research showed the usefulness and effectiveness of mobile-based treatments for cancer care management. The present review provided an in-depth analysis of mobile technology for lifestyle changes in the cancer population. The proposed taxonomy can be used as a starting point for the methodical characterization of mHealth solutions, which despite their wide range, are often described in similar words [97]. This taxonomy is important, as it defines and categorizes the key characteristics of the users’ interaction and engagement in mHealth interventions on cancer patients; therefore, it could be considered in the creation of mobile apps focused on cancer management in the future. In addition, most of the included studies showed good adherence to mHealth interventions, indicating the promising application of mHealth in cancer management.

### 4.3. Study Limitations

First, we were unable to conduct a meta-analysis due to the multiplicity of study designs and efficacy assessments. Second, the methodological issues (small sample size and lack of control groups) deterred the synthesis and assessment of overall evidence strength. Third, as the mean age of the participants from the selected studies varied from 14.2 years to 72 years, we were unable to discuss the implications of elderly people older than 75 years. Fourth, the search results for this study were based on MeSH terms and relevant keywords along with their combinations found in cancer care literature reviews. Fifth, the review was limited to English language articles only. Finally, it did not include any grey literature.

## 5. Conclusions

This research focused on identifying the factors that impact the effectiveness of mobile-based apps and provided a taxonomy classification that explains the design and efficacy of interventions for cancer care treatment and management. Various factors have been identified as facilitators of successful mHealth interventions, including guided supervision, personalized suggestions, a strong theoretical basis, and the use of wearable technology. These elements have been shown to improve both adherence to treatment regimens and the overall efficacy of mHealth interventions. However, despite these positive findings, there are still barriers to the widespread adoption of mHealth, such as technical difficulties with devices and apps, fatigue, and lack of intervention support. Additionally, various factors such as old age, mortality, personal reasons, and unfamiliarity with mobile devices can lead to dropouts from mHealth programs. Therefore, while the benefits of mHealth are clear, further research is needed to address the challenges that may hinder its effectiveness and ensure that mHealth interventions are accessible and useful to a diverse range of individuals. The results highlighted the need for well-designed trials and robust theory-based mHealth interventions in determining the efficacy and impact of mobile health interventions in cancer care. With the advancements and expansion of mHealth technologies, there are increasing opportunities for these mobile apps in personalized health care and behavioural change for cancer patients. For future studies, we highly recommend investigating, improving and verifying this taxonomy classification in order to enhance the efficacy of mHealth interventions for cancer care. These targets are aligned with the goals of participatory health informatics, where the work for defining the types of interventions that foster the participation of patients in their own healthcare is in progress. Furthermore, as per recommendations, future research should look into how to expand this taxonomy to address core aspects of cancer self-management with a holistic approach to the adherence to pharmacological treatments in cancer and addressing the challenges of disease, patient, and socioeconomic factors in the cancer care domain.

## Figures and Tables

**Table 2:** Cancer type, cancer health outcomes, duration and usage rate of mobile-app-based interventions for different cancer types.

Cancer Type (n-Number of Studies)	Positive Outcome(+)	Neutral Outcome	Duration (Min-Max) (Weeks)	Usage Rate (Max-Min) (%)
Breast Cancer (*n* = 20)	19	1	4–24	100–74.5%
Mixed Cancer (*n* = 23)	19	4	4–24	100–25%
Prostate Cancer (*n* = 2)	2	0	8–12	89.4–79%
Lung Cancer (*n* = 2)	2	0	12	100–90%
Pancreatic Cancer (*n* = 2)	1	1	12–32	82.5–79%
Oral Cancer (*n* = 1)	12	0	12	100%
Brain Tumour *(n* = 1)	1	0	8	80%
Aerodigestive Cancer (*n* = 1)	1	0	8	100%
Hematologic Cancer (*n* = 1)	0	1	16	90%
Myeloid Neoplasm *(n* = 1)	0	1	8 to 12	88%
Oesophageal Cancer *(n* = 1)	0	1	8	83.3%

**Table 3:** Distribution of studies based on mobile app usage rate and duration.

Completion/Usage Rate (%)	Study Duration ≤ 12 Weeks	Study Duration 13–32 Weeks
=≥80%	33 studies	8 studies
<80%	10 studies	4 studies
Total studies	43 studies	12 studies

**Table 4:** Taxonomy of the included studies based on four dimensions.

1. Theoretical foundation or behavioural techniques																
BCTs	SCT	CBT	CBSM	CT	LTs	GST	SDT	MI	ACT	CohT	CopT	SST	TAM	GBL	EBT	MBSR
[37,45,56,59,66]	[37,38,43,45,49,54,67]	[62,64,70]	[61]	[37,38,45]	[37,45,60]	[37,45,60]	[27,34]	[60]	[60]	[30]	[30]	[30]	[81]	[46]	[39]	[48]
2. Delivery Mechanisms																
Reminders/Alerts/Push Notifications								Tailored Messages/Lifestyle Recommendations								
[28,31,32,34,37,40,44,45,47,51,52,55,57,58,61,63,69,71,74,81]								[16,27,29,35,36,38,59,65,68,72,73,77,78]								
3. Psycho-educational Program																
[31,40,42,53,54,64,81]																
4. Social Platform																
WeChat						Facebook				WhatsApp				Line App		
[41]						[27,34]				[42]				[75]		

BCTs—behaviour change techniques, SCT—social cognitive theory, CBT—cognitive behavioural theory, CBSM—cognitive behavioural stress management, CT—control theory, LTs—learning theories. GST—goal setting theory, SDT—self-determination theory, MI—motivational interviewing, ACT—acceptance commitment therapy, CohT—coherence theory, CopT—coping theory, SST—social support theory, TAM—science and technology acceptance model, GBL—game-based learning, EBT—evidence-based treatment, and MBSR—mindfulness-based stress reduction.
